# The Association of Episiotomy with Obstetric Anal Sphincter Injury–A Population Based Matched Cohort Study

**DOI:** 10.1371/journal.pone.0107053

**Published:** 2014-09-09

**Authors:** Sari Räisänen, Tuomas Selander, Rufus Cartwright, Mika Gissler, Michael R. Kramer, Katariina Laine, Seppo Heinonen

**Affiliations:** 1 Department of Epidemiology, Rollins School of Public Health, Emory University, Atlanta, Georgia, United States of America; 2 Department of Obstetrics and Gynaecology, Kuopio University Hospital, Kuopio, Finland; 3 Department of Science Service Center, Kuopio University Hospital, Kuopio, Finland; 4 Department of Epidemiology & Biostatistics, Imperial College London, London, United Kingdom; 5 National Institute for Health and Welfare (THL), Helsinki, Finland, Nordic School of Public Health, Gothenburg, Sweden; 6 Department of Obstetrics, Oslo University Hospital, Ullevål, Norway, Faculty of Medicine, University of Oslo, Oslo, Norway; 7 School of Medicine, University of Eastern Finland, Kuopio, Finland; Indiana University School of Medicine, United States of America

## Abstract

**Objectives:**

To estimate the independent association of episiotomy with obstetric anal sphincter injuries (OASIS) using first a cross-sectional and then a matched pair analysis.

**Design:**

A matched cohort.

**Setting:**

Data was gathered from the Finnish Medical Birth Register from 2004–2011.

**Population:**

All singleton vaginal births (n = 303,758).

**Methods:**

Women resulting matched pairs (n = 63,925) were matched based on baseline risk of OASIS defined based on parity (first or second/subsequent vaginal births), age, birth weight, mode of delivery, prior caesarean section, and length of active second stage of birth.

**Results:**

In cross-sectional analysis episiotomy was associated with a 12% lower incidence of OASIS (adjusted odds ratio (aOR) 0.88, 95% confidence interval (CI) 0.80 to 0.98) in first vaginal births and with a 132% increased incidence of OASIS in second or subsequent vaginal births (aOR 2.32, 95% CI 1.77 to 3.03). In matched pair analysis episiotomy was associated with a 23% (aOR 0.77, 95% CI 0.69 to 0.86) lower incidence of OASIS in first vaginal births and a 61% (aOR 1.61, 95% CI 1.14 to 2.29) increased incidence of OASIS in second or subsequent vaginal births compared to women who gave birth without an episiotomy. The matched pair analysis showed a 12.5% and a 31.6% reduction in aORs of OASIS associated with episiotomy, respectively.

**Conclusions:**

A matched pair analysis showed a substantial reduction in the aORs of OASIS with episiotomy, due to confounding by indication. This indicates that results of observational studies evaluating an association between episiotomy and OASIS should be interpreted with caution.

## Introduction

An association between obstetric anal sphincter injuries (OASIS) and episiotomy has been a topic of on-going debate with highly heterogeneous results of previous studies. A previous systematic review of randomized controlled trials supported restrictive use of mediolateral or midline episiotomy, since these techniques result in a higher incidence of severe perineal trauma [1]. However, results of previous observational studies have been conflicting, with reports of large positive (harm) and large negative associations (benefit) [2]–[6]. Such differences might be explained by differences in obstetric practices, such as episiotomy angle or perineal protection techniques. Equally though, these observational studies may be biased by unmeasured factors, including particularly the indication for episiotomy. Confounding by indication may occur whenever the indication for intervention is to reduce the risk for prospective outcome [7]; in the present context it means that episiotomy (intervention) is reserved for the patients with a higher risk of OASIS (prospective health outcome) to reduce the risk of OASIS. The likelihood of confounding by indication increases, if differences in groups receiving and not receiving the intervention are observed [8]. Utilizing the same population based data, gathered from the Finnish Medical Birth Register (MBR), we previously demonstrated that episiotomy was performed more frequently for women at higher baseline risk of OASIS, compared to women giving birth without an episiotomy [9]. Over the time period 2004–2011 while episiotomy use decreased, this disparity in baseline risk of OASIS increased. Paradoxically over this time period we observed a crossover in the association between episiotomy and OASIS. The previously observed negative association between episiotomy and OASIS, changed to a positive association due to increased confounding by indication, as clinicians increasingly used episiotomy selectively for women at higher baseline risk of OASIS [9].

The aim of our study was to estimate the independent association of episiotomy with OASIS. We conducted a cross-sectional analysis to compare all women with and without OASIS, and to minimise confounding by indication. We further conducted a matched-pair analysis of women with and without episiotomy matched on their baseline risks of OASIS.

## Materials and Methods

### Data and population

The data were gathered from two national health registers, the MBR and the Hospital Discharge Register (HDR) currently maintained by the National Institute for Health and Welfare (THL). Permission to use the confidential register data in this study was approved on 16th February, 2012 by the National Institute for Health and Welfare (THL) in Finland. (Reference number 1749/5.05.00/2011). No informed consent was needed since data was anonymous register based information and comprised the total population.

The MBR includes information on maternal and neonatal birth characteristics and perinatal outcomes during first postnatal week for all births including stillbirths after the 22nd gestational week or weighing 500 g or more. The HDR provides information on inpatient and outpatient care such as diagnoses, medical interventions and surgical procedures. Using an encrypted unique personal identification numbers the two data sources were linked together.

The dataset included 303,758 women with singleton vaginal first births during the study period from 2004 to 2011. We excluded women who gave birth by CS (n = 73,402), did not have information on length of the active second stage of the birth (n = 83,984), or had a second or subsequent OASIS (n = 34). Using the remaining population we identified 63,925 matched pairs of women who gave birth with and without episiotomy with an equal baseline risk of OASIS. Each woman with episiotomy was matched to a woman without episiotomy and exactly same risk profile of OASIS based on categorical maternal age groups (<20, 20–29, 30–39 or ≥40), categorical birth weight groups (<3000 g, 3000–3499 g, 3500–3999 g or ≥4000 g), mode of delivery (spontaneous vaginal, breech, forceps or vacuum assisted), prior CS (yes or no), and categorical length of active second stage of birth (≤30 min, 31–69 min, 70–169 min or ≥170 min). The matching was done separately for women with first vaginal births and women with second or subsequent vaginal births. Women with first vaginal births were analysed together with women admitted for first vaginal births after a previous caesarean section (CS).

### Variables and definitions

The degree of OASIS was diagnosed by physicians and classified by the standard definition of perineal tears: in a third degree tear, either external or both external and internal anal sphincter muscles are injured, in a fourth degree tear, both the anal sphincter muscles and the anorectal mucosa are injured [10]. OASIS cases registered according to the International Classification of Diseases (ICD-10) codes O70.2 (3^rd^ degree) and O70.3 (4^th^ degree) were derived from the MBR. The OASIS diagnosis was double-checked/cross-checked by collecting the information of OASIS surgical primary repair from the HDR, and no other information was gathered from the HDR. Third and fourth degree injuries were pooled for all the analyses. Mode of delivery was classified as either vaginal spontaneous, breech, forceps or vacuum assisted. Active second stage of labour was defined as commencement of active pushing until birth of the infant. The exclusive practice in Finland is of lateral episiotomy type [11], contrasting with more common use of mediolateral or midline episiotomy in other parts of the world.

### Statistical analyses

Differences in demographics and delivery characteristics between women with and without OASIS, and with and without episiotomy, were assessed by Chi Square or Mann Whitney U tests. An association between OASIS and episiotomy was first determined by traditional logistic regression analysis separately for first vaginal births and for the second or subsequent vaginal births by using women without OASIS as a reference group. Odds ratios (ORs) of OASIS with 95% confidence intervals (CI) were adjusted for maternal age, birth weight, mode of delivery, prior CS, episiotomy, and length of active second stage of labour that are known risk factors for OASIS based on previous analyses with the same data. Furthermore, in order to estimate confounding by indication in the cross-sectional analysis we matched pairs of women who gave birth with or without episiotomy with equal risk of OASIS as described previously. ORs of OASIS with 95% CIs were determined for matched pairs by conditional logistic regression analysis. The data were analyzed using SPSS 19.0 and R statistical software version 2.15.2.

## Results

OASIS was associated with advanced maternal age, higher birth weight, vacuum assisted birth, and an episiotomy regardless of vaginal birth order (Table 1). An episiotomy was performed more frequently among women with advanced maternal age (≥30 years), high birth weight, delivery assisted by vacuum extraction, and prolonged active second stage of labour regardless of vaginal birth order (Table 2). Among first vaginal births OASIS incidence among women with episiotomy was 2.3% and 1.0% among women without episiotomy, and 0.6% and 0.2%, respectively among second or subsequent vaginal births.

**Table 1:** Demographics and delivery characteristics separately among women with first vaginal singleton births including women with first vaginal birth after a prior caesarean section and second or subsequent vaginal births with and without OASIS from 2004–2011 in Finland.

	First vaginal birth, n = 131,006			Second or subsequent vaginal birth, n = 172,752		
Demographic/delivery characteristic	With OASIS,	Without OASIS,	p value*	With OASIS,	Without OASIS,	p value*
	n = 1,866	n = 129,140		n = 367	n = 172,385	
	(1.4%)	(98.6%)		(0.2%)	(99.8%)	
Mean maternal age, years (SD)	28.3 (4.7)	27.1 (5.1)	≤0.001	31.4 (4.8)	30.6 (5.0)	≤0.001
Maternal age, years %			≤0.001			0.001
≤19	2.3	5.6		3.0	8.9	
20–29	57.4	61.0		17.7	29.5	
30–39	39.1	31.9		37.3	39.7	
≥40	1.2	1.5		42.0	21.8	
Mean birth weight, g (SD	3674 (442)	3444 (487)	≤0.001	3908 (514)	3625 (495)	≤0.001
Birth weight, g %			≤0.001			≤0.001
≤2999	5.7	15.7		3.1	9.2	
3000–3499	29.4	37.9		17.6	29.7	
3500–3999	42.0	34.7		37.3	39.6	
≥4000	22.9	11.6		42.0	21.6	
Mode of delivery %			≤0.001			≤0.001
Vaginal spontaneous	69.0	80.9		87.1	96.8	
Breech	0.4	0.8		0.6	0.6	
Forceps	0.6	0.1		0.0	0.0	
Vacuum assistance	30.0	18.2		12.2	2.6	
A prior caesarean section %	13.0	8.3	≤0.001	7.3	5.6	0.10
Episiotomy %	64.6	51.8	≤0.001	36.1	7.7	≤0.001
Mean length of active second stage, min. (SD)	60.2 (57)	47.8 (51)	≤0.001	29.1 (35)	13.8 (21)	≤0.001
Length of active second stage, min. %			≤0.001			≤0.001
≤30	37.7	50.6		72.5	91.6	
31–69	35.1	30.5		18.8	6.4	
70–169	23.3	16.5		7.9	1.8	
≥170	3.9	2.6		0.8	0.2	

*Chi Square or Mann Whitney U –test.

**Table 2:** Demographics and delivery characteristics among women with first vaginal singleton births including women with first vaginal births after a prior caesarean section and among women with second or subsequent vaginal births with and without episiotomy from 2004–2011 in Finland.

	First vaginal birth, n = 131,006			Second or subsequent vaginal birth, n = 172,752		
Demographic/delivery characteristic	With episiotomy,	Without episiotomy,	p value*	With episiotomy,	Without episiotomy,	p value*
	52.3%	47.7%		7.6%	92.4%	
OASIS %	2.3	1.0	≤0.001	0.6	0.2	≤0.001
Mean maternal age, years (SD)	27.7 (5.1)	27.1 (5.2)	≤0.001	31.1 (5.0)	30.6 (5.0)	≤0.001
Maternal age, years %			≤0.001			≤0.001
≤19	5.4	6.6		0.3	0.4	
20–29	62.4	64.8		38.6	42.7	
30–39	30.9	27.5		56.2	52.5	
≥40	1.4	1.1		4.9	4.4	
Mean birth weight, g (SD)	3494 (481)	3386 (507)	≤0.001	3701 (513)	3619 (494)	≤0.001
Birth weight, g %			≤0.001			≤0.001
≤2999	13.6	17.7		7.2	9.0	
3000–3499	35.8	39.9		26.0	29.8	
3500–3999	36.6	32.9		39.4	39.8	
≥4000	14.0	9.4		27.4	21.4	
Mode of delivery %			≤0.001			≤0.001
Vaginal spontaneous	69.6	92.9		87.2	96.8	
Breech	1.3	0.3		0.3	0.6	
Forceps	0.2	0.0		0.0	0.0	
Vacuum assistance	29.8	6.8		12.5	2.6	
A prior caesarean section %	8.3	8.5	0.95	7.4	5.7	0.16
Mean length of active second stage, min. (SD)	51.9 (54)	43.7 (48)	≤0.001	23.7 (32)	13.1 (19)	≤0.001
Length of active second stage, min. %			≤0.001			≤0.001
≤30	46.6	54.6		72.5	91.6	
31–69	31.5	29.5		18.8	6.4	
70–169	19.1	13.7		7.9	1.8	
≥170	3.9	2.5		0.8	0.2	

*Chi Square or Student’s t -test.

After adjustment for maternal age, birth weight, mode of delivery, prior CS, episiotomy and length of active second stage of labour in traditional logistic regression analysis, episiotomy was associated with a 12% lower incidence of OASIS (adjusted ORs 0.88, 95% CI 0.80 to 0.98) among women with first vaginal birth. Among parous women, with second or subsequent vaginal births, a 132% increased incidence of OASIS was observed in deliveries with episiotomy (aOR 2.32, 95% CI 1.77 to 3.03) compared with parous women without an episiotomy (Table 3). Table 4 shows delivery characteristics among matched pairs for both vaginal birth order groups. Among matched first vaginal births, incidence of OASIS among women with episiotomy was 1.1%, and 1.4% among women without episiotomy. Among matched second or subsequent vaginal births OASIS incidence with and without episiotomy was 0.6% and 0.4%, respectively. Among matched pairs of women, episiotomy was associated with a 23% (aOR 0.77, 95% CI 0.69 to 0.86) lower incidence of OASIS in first vaginal births, and a 61% (aOR 1.61, 95% CI 1.14 to 2.29) increased incidence of OASIS in second or subsequent vaginal births than women without an episiotomy (Table 5).

**Table 3:** Adjusted odds ratios (aOR) of OASIS among women with first vaginal singleton births including women with first vaginal birth after a prior caesarean section (n = 131,006) and among women with second or subsequent births (n = 172,710) in 2004–2011 in Finland (logistic regression analysis).

	First vaginal birth	Second or subsequent vaginal birth
Demographic/delivery	aOR (95% CI)	aOR (95% CI)
characteristic		
OASIS, N	1,866	367
Maternal age, year		
≤19	1	
20–29 (≤29 ref multiparous)	1.90 (1.42–2.53)	1
30–39	2.29 (1.71–3.08)	1.32 (0.19–9.45)
≥40	2.06 (1.28–3.31)	1.33 (0.18–9.95)
Birth weight, g		
≤2999	1	1
3000–3499	2.03 (1.65–2.50)	1.69 (0.89–3.21)
3500–3999	2.99 (2.43–3.67)	2.45 (1.32–4.54)
≥4000	4.67 (3.76–5.80)	4.53 (2.45–8.38)
Mode of delivery		
Vaginal spontaneous	1	1
Breech	0.87 (0.43–1.75)	0.47 (0.07–3.37)
Forceps	4.88 (2.70–8.82)	NA
Vacuum assistance	1.68 (1.50–1.87)	2.03 (1.41–2.90)
A prior caesarean section	1.39 (1.20–1.59)	1.19 (0.80–1.76)
Episiotomy	0.88 (0.80–0.98)	2.32 (1.77–3.03)
Length of active second stage, min.		
≤30	1	1
31–69	1.32 (1.18–1.47)	2.51 (1.90–3.33)
70–169	1.37 (1.21–1.55)	3.05 (2.01–4.62)
≥170	1.42 (1.11–1.82)	2.99 (0.94–9.50

CI = confidence interval, NA = not applicable.

**Table 4:** Demographics and delivery characteristics for women with matched baseline risk of OASIS with and without episiotomy separately for women with first vaginal singleton births including women with first vaginal birth after a prior caesarean section and second or subsequent vaginal births in 2004–2011 in Finland.

Demographic/delivery characteristic	First vaginal birth		Second or subsequent vaginal birth	
	with episiotomy	without episiotomy	with episiotomy	without episiotomy
Pairs, n	50,897		13,028	
OASIS, N (%)	548 (1.1)	707 (1.4)	82 (0.6)	51 (0.4)
Mean maternal age, years (SD)	27.0 (5.0)	26.9 (5.1)	31.1 (5.0)	31.0 (5.0)
Maternal age, years %				
≤19	5.9	5.9	0.3	0.3
20–29	64.3	64.3	38.6	38.6
30–39	28.7	28.7	56.3	56.3
≥40	1.2	1.2	4.8	4.8
Mean birth weight, g (SD)	3459 (478)	3447 (485)	3702 (512)	3688 (502)
Birth weight, g %				
≤2999	14.8	14.8	7.1	7.1
3000–3499	37.4	37.4	26.0	26.0
3500–3999	36.4	36.4	39.4	39.4
≥4000	11.4	11.4	27.5	27.5
Mode of delivery %				
Vaginal spontaneous	91.3	91.3	74.4	74.4
Breech	0.3	0.3	14.5	14.5
Forceps	0.0	0.0	0.0	0.0
Vacuum assistance	8.4	8.4	14.0	14.0
A prior caesarean section %	8.4	8.4	6.5	6.5
Mean length of active second stage, min. (SD)	45.3 (47)	45.2 (47)	23.2 (30)	20.4 (29)
Length of active second stage, min. %				
≤30	51.6	51.6	80.2	80.2
31–69	31.3	31.3	14.5	14.5
70–169	15.1	15.1	4.9	4.9
≥170	2.0	2.0	0.4	0.4

SD = standard deviation.

**Table 5:** Adjusted odds ratios (aOR) of OASIS for matched pairs of women with and without episiotomy separately for women with first vaginal singleton birth including women with first vaginal birth after a prior caesarean section and among women with second or subsequent birth from 2004–2011 in Finland (logistic regression analyses).

	First vaginal birth	Second or subsequent vaginal birth
Demographic/delivery	aOR (95% CI)	aOR (95% CI)
characteristic		
OASIS, N	1,255	133
Maternal age, years		
≤19	0.58 (0.41–0.81)	
20–29 (≤29 ref. multiparous)	1	1
30–39	1.29 (1.14–1.46)	1.64 (1.11–2.43)
≥40	1.60 (1.04–2.47)	1.21 (0.51–2.91)
Birth weight, g		
≤2999	1	1
3000–3499	2.09 (1.61–2.71)	4.65 (0.62–34.78)
3500–3999	3.05 (2.36–3.94)	8.12 (1.12–58.85)
≥4000	4.84 (3.69–6.34)	11.40 (1.57–82.70)
Mode of delivery		
Vaginal spontaneous	1	1
Breech	0.87 (0.22–3.51)	0.48 (0.07–3.45)
Forceps	10.77 (4.16–27.89)	NA
Vacuum assistance	1.23 (1.03–1.46)	1.48 (0.97–2.25)
A prior caesarean section	1.46 (1.24–1.73)	1.07 (0.56–2.04)
Episiotomy	0.77 (0.69–0.86)	1.61 (1.14–2.29)
Length of active second stage, min.		
≤30	1	1
31–69	1.41 (1.24–1.61)	2.10 (1.38–3.19)
70–169	1.63 (1.39–1.90)	3.09 (1.80–5.28)
≥170	1.83 (1.34–2.50)	3.64 (0.87–15.30)

CI = confidence interval, NA = not applicable.

Comparing the results of the matched pair analysis to the results of the cross-sectional analysis caused a 12.5% and a 31.6% relative reduction in aORs of OASIS associated with episiotomy in first and second or subsequent births, respectively, with this difference being due to reduction in residual confounding.

## Discussion

### Main findings

Traditional regression approaches to control for confounding by indication of the association between episiotomy and OASIS may be inefficient, and produce biased estimates with residual confounding. By employing cohort-matching approach we demonstrate further bias reduction due to confounding by indication and other unmeasured factors. In the present study, the aim was to estimate the unbiased association between episiotomy and OASIS, net of factors which are both indications for episiotomy and risk factors for OASIS, by comparing traditional regression and matched pair analysis of birth cohort data. Women who underwent an episiotomy represented a high-risk population by virtue of use of vacuum extraction, higher birth weights, or prolonged active second stage of birth compared with women who gave birth without an episiotomy. In the matched pair analysis, women with episiotomy were matched with women without episiotomy based on age, birth weight, mode of delivery, prior CS and length of active second stage of labour, to reduce the difference in baseline risk of OASIS between women who gave birth with and without episiotomy. Pair matching exposed and unexposed women limited confounding by indication, and reduced bias in effect estimates as compared to traditional regression approaches (difference in OR of 12.5% and a 31.6% in first vaginal births and second or subsequent vaginal births, respectively).

### Strengths and limitations

The present study has several strengths. The data was derived from the two national health registers with high-quality information and good coverage [12], [13], and it covered the most recent time period unlike previous studies [2], [14], [15]. The most important strength of the present study was that to the best of our knowledge this was the first large population based study where women giving birth with and without episiotomy were matched based on delivery characteristics and baseline risk of OASIS. The possible limitation was that we did not have information on all known risk factors for OASIS such as abnormal presentation. Further, in first vaginal births matched pairs did not include all OASIS cases and included fewer cases especially at higher OASIS risk such as women giving birth by vacuum extraction, since most of them underwent an episiotomy, and we were not able to find a pair for all women with OASIS.

### Interpretation

In the present cohort study, as a result of matching, in first vaginal births we observed a 12.5% increase in the estimated “protective” role of episiotomy, whereas in second or subsequent births we observed a 31.6% reduction in the estimated harm associated with episiotomy. It indicated that traditional regression analysis might be inefficient to control for confounding by indication. However, our data lacked information on the several actual episiotomy indications such as slow crowning, obstructed labour, relative maternofetal disproportion, abnormal fetal head presentation and imminent perineal rupture. Therefore there still might be residual confounding of the cases especially among women with second or subsequent vaginal births with low use of episiotomy and very low incidence of OASIS. Our results indicates that observational study design is susceptible to several kind of bias such as confounding, information and selection bias as showed in several previous studies [8], [16]–[18], and benefits or harms associated with episiotomy, are due to its indications for use, and not solely due to its treatment effects.

Randomized controlled trials (RCTs) are the gold standard for evaluating effectiveness of medical interventions such as episiotomy. In RCTs independent variables such as mode of delivery, birth weight and parity should be exchangeable between exposed and unexposed women. However, confounding by indication may still influence the outcome of RCTs where clinicians may influence the risk of receiving the intervention after randomisation. Such an effect has indeed been reported in an RCT of restrictive vs. routine midline episiotomy [19]. Based on our data very large sample sizes would be required in any RCT, even for high risk women. We estimate that an adequate sample size for women with first vaginal births giving birth by vacuum extraction with an infant weighing 4000 grams or more would be 2,566 cases for 80% power. The same numbers for women with first spontaneous vaginal births with an infant weighing up to 3500 g would be much higher at 11,610 cases. This kind of study design, however, contains ethical concerns and probably would not be allowed to be implemented. Most previous RCTs have compared only restrictive and routine use of episiotomy – requiring an even larger sample size – and have either used an inadequate sample size, [20]–[24] or had unexpected differences in independent variables between the groups [22] suggesting random error. Randomization does not guarantee that treatment and control group will have the same risk of the outcome especially if there are unknown risk factors or an inadequate sample size is used.

### Conclusions

In the present study, we demonstrated a substantial influence of confounding by indication on the observed association of episiotomy and OASIS. Many current indications for episiotomy, relating to signs of obstructed labour, are also risk factors for OASIS, which contributes to the higher unadjusted incidence of OASIS among women who give birth with episiotomy. Although results of observational studies evaluating associations between episiotomy and OASIS should therefore be interpreted with caution. After careful matching, our results suggest a substantial protective benefit of lateral episiotomy at first vaginal births. Further randomized trials are still needed to evaluate the indications and techniques for episiotomy among women with different baseline risks of OASIS.
